# First Characterization of a Cluster of VanA-Type Glycopeptide-Resistant *Enterococcus faecium,* Colombia

**DOI:** 10.3201/eid0809.10.3201/eid0809.010435

**Published:** 2002-09

**Authors:** Diana Panesso, Sigifredo Ospina, Jaime Robledo, María Claudia Vela, Julieta Peña, Orville Hernández, Jinnethe Reyes, César A. Arias

**Affiliations:** * Universidad El Bosque, Bogotá, Colombia; † Hospital San Vicente de Paul, Medellín, Colombia; ‡ Centro de Investigaciones Biológicas, Medellín, Colombia; § Instituto Nacional de Salud, Bogotá, Colombia

**Keywords:** vancomycin, enterococci, resistance, glycopeptide-resistant enterococci

## Abstract

From August 1998 to October 1999, glycopeptide-resistant enterococci (GRE) were isolated from 23 infected patients at a teaching hospital in Medellín, Colombia. Identification at the species level and by multiplex polymerase chain reaction assay indicated that all isolates were *Enterococcus faecium*. The isolates were highly resistant to ampicillin, ciprofloxacin, gentamicin, penicillin, streptomycin, teicoplanin, and vancomycin; they were susceptible only to chloramphenicol, linezolid, and nitrofurantoin. Determination of glycopeptide genotype indicated the presence of the *van*A gene in all isolates. Molecular typing by pulsed field gel electrophoresis showed that all isolates were closely related. This study is the first molecular characterization of GRE in Colombia.

Enterococci normally colonize the intestinal tract of humans and other animals, with urinary tract infection being the most common enterococcal infection reported in humans (1). In recent years, enterococci have become important nosocomial pathogens: the organisms have been reported as the second leading cause of urinary tract infections and the third leading cause of nosocomial bacteremia in hospitalized patients (2). The most commonly identified species is *Enterococcus faecalis,* followed by *E. faecium* (3). *E. gallinarum*, *E. casseliflavus*, and *E. durans* have been reported less often (4,5).

 The most important characteristics of these organisms include their inherent resistance to several antimicrobial agents and their ability to acquire resistance determinants. Resistance against such diverse groups of drugs as β-lactams, macrolides, aminoglycosides, and glycopeptides continues to evolve. The ability to grow in the presence of glycopeptides results from the change of the C-terminal residue of peptidoglycan precursors (D-Ala) to D-lactate (VanA, VanB, and VanD phenotypes) (6,7) or D-serine (VanC, VanE, and VanG phenotypes) (8–10). The change alters the affinity of the glycopeptide for its natural target (6). Six different gene clusters have been described (*van*A-B-C-D-E-G) (6,10–12). The most predominant phenotype in *E. faecium* is VanA; VanA strains are highly resistant to both vancomycin and teicoplanin. The *vanA* gene cluster is located on transposons or related elements (6) and has also been found in nonenterococcal species such as *Arcanobacterium* (*Corynebacterium*) *haemolyticum*, *Oerskovia turbata*, *Bacillus circulans*, and *Streptococcus gallolyticus* (13–16). A *van* cluster with a high degree of homology to the *van*A cluster (designated *vanF*) has been found in the biopesticide organism *Paenibacillus popilliae* (17).

Since the initial discovery of glycopeptide-resistant enterococci (GRE) in the United Kingdom (18), nosocomial isolates of GRE have been reported from around the world (14); these isolates have also been found in healthy people in the community outside the hospital (19). In Latin America, GRE have been reported in Argentina (20) and Brazil (21). We report here the first isolation and characterization of a cluster of VanA-type glycopeptide-resistant *E. faecium* in a teaching hospital in Colombia.

## Materials and Methods

### Bacterial Isolates

Hospital San Vicente de Paul is a 650-bed teaching hospital providing tertiary care for Medellín, Colombia, and neighboring towns, an area with a population of 1.5 million inhabitants. From August 1998 to October 1999, we collected organisms from 23 patients. Enterococci were isolated from infected patients by classical microbiologic techniques (3). Identification at the species level was performed by the Vitek gram-positive card (bioMérieux SA, Marcy l'Etoile, France), according to the manufacturer’s recommendations.

### Antimicrobial Susceptibility Testing

Initial identification of resistance to vancomycin was performed by the Vitek system (bioMérieux SA). We confirmed resistance to vancomycin, determining MICs by an agar dilution method as recommended by the National Committee for Clinical Laboratory Standards (22) on Mueller-Hinton agar plates (ICN Pharmaceuticals Inc., Madison, WI). MICs were performed in duplicate. The following antimicrobial agents were obtained as reference powders of known potency and tested: ampicillin, ciprofloxacin, chloramphenicol, gentamicin, penicillin, streptomycin, teicoplanin, vancomycin (ICN Pharmaceuticals, Inc.), and linezolid (Pharmacia Corp., Peapack, NJ). Susceptibility to nitrofurantoin (MIC <32 µg/mL) was determined by the Vitek system (bioMérieux SA). In addition to determining MICs, high-level resistance to streptomycin was tested at concentrations of 2 mg/mL; *E. faecalis* ATCC 29212 was used as control strain. Three well-characterized strains of enterococci belonging to the genotypes *van*A (*E. faecium* BM4147), *van*B (*E. faecalis* V583), and *van*C (*E. gallinarum* BM4174) were included as GRE control strains.

### Polymerase Chain Reaction (PCR) for Species Identification of Enterococci and the *van* Genes

For species identification of enterococcal isolates, the genes encoding D-alanine-D-alanine ligases specific for *E. faecium* (*ddl_E. faecium_*), *E. faecalis* (*ddl_E. faecalis_*), *van*C-1 (*E. gallinarum*), and *van*C-2 (*E. casseliflavus*) were detected by a multiplex PCR assay, as described by Dutka-Malen et al. (23). Primers D1 (5´ GCTTCTTCCTTTACGACC) and D2 (GTTCCAGTCCTAAAAAAC) for the *ddl* gene of *E. avium* were included in the multiplex mixture. A similar multiplex PCR protocol was performed separately for detection of *van* genes by using specific primers for *van*A, *van*B, *van*C-1, and *van*C-2 genes (23). *E. faecium* BM4147 (*van*A), *E. faecalis* V583 (*van*B), and *E. gallinarum* BM4174 (*van*C-1) were used as control strains.

### Genotyping

Molecular typing was performed by pulsed-field gel electrophoresis (PFGE). Chromosomal DNA was obtained by the procedure of Antonishyn et al. (24): a loopful of bacterial colonies from a 24-h isolate was grown until *A*_600_ was 0.1 in brain heart infusion broth at 37°C. Bacteria were harvested by centrifugation at 4°C, and the pellet was resuspended in cell suspension buffer (1M NaCl, 10 mM Tris-HCl, pH 8.0). The suspension was embedded in 1.5% agarose and disks were made. Disks were placed in lysis buffer (6mM Tris-HCl, pH 8, 1 M NaCl, 100 mM EDTA, 0.5% Brij-58, 0.2% Na deoxycholate, and 0.5% N-lauroyl sarcosine) with additional RNase (20 µg/mL) and lysozyme (1 mg/mL) and incubated for 4 h at 37°C. The disks were washed with EDTA-sarcosine buffer (0.5 M EDTA, pH 8, and 0.1% N-lauroyl sarcosine), placed in proteinase K solution (100 µg/mL), and incubated overnight at 50°C with mild agitation. Disks were washed four times with Tris-EDTA buffer (Tris 10 mM, pH 7.5, and 1mM EDTA) for 30–60 min at room temperature on a rocker.

DNA was digested as described (25). Briefly, DNA fixed in the agarose disks was preincubated in 1 mL of buffer E (6 mM Tris, pH 8, 20 mM KCl, 6 mM MgCl_2_, and 6 mM 2-mercaptoethanol) at 25°C for 30 min. Restriction was performed for 17 h in 60 µL of restriction buffer containing *Sma*I (20 U) at 25ºC. The reaction was stopped by addition of 10 µL of sterile loading buffer. Gels were prepared with 1% agarose in 0.5x TBE buffer (50 mM Tris, pH 8, 50 mM boric acid, 0.2 mM EDTA). A DNA ladder (50–1000 kb) was used as the molecular size marker. Fragments were separated by electrophoresis (CHEF-DR II system, Bio-Rad Laboratories, Inc., Richmond, CA) at 6 V/cm, with switch times ramped from 1 s to 35 s over 23 h at 14°C. After staining with ethidium bromide, the restricted DNA fragments were viewed under UV light and photographed. A vancomycin-susceptible strain of *E. faecium* isolated in the same hospital was included in the PFGE protocol as the control. We interpreted the band patterns by the criteria of Tenover et al. (26).

## Results

### GRE Isolates and Identification

From August 1998 to October 1999, 23 GRE were collected from the same number of patients hospitalized in various wards in Hospital San Vicente. The first isolate was recovered from the pleural fluid of a patient hospitalized in the surgical ward. Isolates came from urine (35%), peritoneal fluid (22%), surgical wound (17%), intra-abdominal abscess (13%), pleural fluid (9%), and bile (4%). Molecular identification by PCR showed that all isolates were *E. faecium*, in agreement with the results of the Vitek gram-positive identification card (bioMérieux SA).

### Antimicrobial Susceptibility Testing

All isolates had high levels of resistance to ampicillin (MICs 128–256 µg/mL), ciprofloxacin (>32 µg/mL), gentamicin (1,024 µg/mL), penicillin (256–512 µg/mL), streptomycin (>2,000 µg/mL), teicoplanin (>32 µg/mL), and vancomycin (512 µg/mL). The isolates were susceptible to chloramphenicol (4–8 µg/mL), linezolid (1 µg/mL), and nitrofurantoin (<32 µg/mL).

### PFGE and Glycopeptide-Resistant Genotype

Analysis of PFGE patterns obtained with the 23 *E. faecium* isolates showed that 21 isolates had the same banding pattern. The remaining two isolates had an additional band around 242 kb (Figure, lanes 2 and 15), indicating that all isolates were closely related (26). This finding suggests the presence of a bacterial clone spreading through different wards during the period of the study. The *vanA* gene was detected in all isolates, in agreement with the antimicrobial susceptibility tests (high-level resistance to both vancomycin and teicoplanin).

**Figure:**
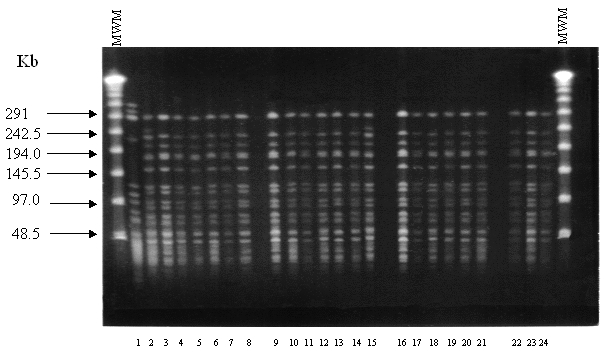
Pulsed-field gel electrophoresis restriction fragment patterns of *Sma*I-digested genomic DNA obtained from glycopeptide-resistant *Enterococcus faecium* isolated at San Vicente de Paul Hospital, Bogotá, Colombia. Lane 1: a susceptible isolate of *E. faecium*; lane 2–24: Restriction patterns of the 23 VanA-type *E. faecium*. MWM, molecular weight marker.

## Discussion

The emergence of multiresistant GRE is a serious nosocomial problem with important implications for hospital infection control. Although the geographic distribution of GRE is worldwide, the epidemiology appears to differ within and across regions. For example, isolates from hospitalized patients in France were shown to be genetically unrelated (27,28); a similar situation has been documented in the United Kingdom (29). In a study of >1,000 isolates of GRE in the United Kingdom, most were *E. faecium* and the VanA phenotype, accounting for 88% of all isolates (30). Although PFGE showed a marked genetic diversity within strains, a common clone was demonstrated in 16 hospitals. In Europe, sources outside hospitals were confirmed as the source of GRE: clonally related vancomycin-resistant enterococci strains have been identified in patients, farm animals, animal products, and the environment, including the presence of GRE in raw meat for human consumption (31–34). Avoparcin, a glycopeptide administered as a growth promoter to farm animals in Europe from 1975 to 2000 (when it was withdrawn from the market), has been implicated as an important factor for the emergence of GRE (31,35). In the United States, dissemination of clonally related strains of GRE was commonly seen in the early stages of the epidemic (14). However, a diverse set of strains has emerged (36). The increased prevalence of GRE in the United States appears to be related to the massive use of vancomycin in hospitals, which by far exceeds the use in Europe (37).

GRE have been found in other parts of Latin America (Argentina and Brazil) (20,21). Results from the SENTRY Antimicrobial Surveillance Program 1997–1999 (38) indicated a low incidence of GRE in Latin America; of 367 isolates, only three had resistance to glycopeptides (two belonged to the VanA phenotype and one VanC-type) (38). This report describes the first characterization of GRE in Colombia; our findings indicate that GRE are emerging as important nosocomial pathogens there. In fact, GRE have now become prevalent in Hospital San Vicente de Paul, and dissemination of isolates to other hospitals in the country is likely. A multicentric surveillance study carried out in 14 teaching hospitals (including five major Colombian cities) from March 2001 to March 2002 indicated that GRE have also been detected in other hospitals, mainly in the capital city of Bogotá, with a prevalence of 10% among clinical isolates of enterococci. Phenotypic characterization demonstrated the presence of both VanA and VanB isolates (39). Of VanA-*E. faecium*, only four had resistance patterns identical to the Medellín isolates described in this study. Genotypic characterization of these isolates is currently under way.

PFGE analysis of the isolates strongly suggests the dissemination of a single clone among hospitalized patients: the emergence of GRE in Colombia is likely to follow a trend similar to the one in the United States. These data may be signaling the start of an epidemic. Factors directly related to the emergence of GRE in Colombia have not been studied properly; glycopeptides appear to be widely used in teaching hospitals, and this situation might be related to the increasing prevalence of methicillin-resistant *Staphylococcus aureus* in the last 4 years (40). Little is known about the use of antimicrobial compounds in animals for human consumption.

Strategies to control the spread of GRE in Hospital San Vicente included monitoring the stringent use of vancomycin and third-generation cephalosporins, providing education to personnel throughout the hospital (especially critical-care units), and implementing infection control measures according to the Hospital Infection Control Practices Advisory Committee (41), strongly emphasizing early detection by the microbiology laboratory of patients colonized or infected with GRE. With these measures, we have decreased the incidence of cases. However, we have not achieved total eradication; in 2001, the prevalence of GRE was 15%.

Resistance of enterococci to multiple antibiotics is common, making treatment problematic. Studies suggest that enterococci inhibited in vitro by <64 µg/mL of ampicillin may be susceptible in vivo to high-dose ampicillin or therapy with ampicillin-sulbactam and gentamicin (if the isolate does not exhibit high-level resistance to gentamicin) (36). However, the isolates from this study exhibited high-level resistance to ampicillin (MIC 128 µg/mL), gentamicin (>1,000 µg/mL), and streptomycin (>2,000 µg/mL), which further limits the therapeutic alternatives. Ciprofloxacin is an antibiotic that has been used as an alternative for the treatment of GRE infections (42), but it was inactive against the isolates examined here.

As found by others (42–44), chloramphenicol was one of the two agents that retained in vitro activity against GRE in this investigation. In a retrospective study of 14 patients with clinical responses, 57% showed improvement after treatment with chloramphenicol (43). Microbiologic response was 73% in 11 patients evaluated in the same study (43). Although no lasting adverse effect related to use of the drug occurred, treatment with chloramphenicol was discontinued for two patients because of chloramphenicol-induced bone marrow suppression (43). In another study of 51 patients with bloodstream infection due to vancomycin-resistant *E. faecium*, 61% and 79% showed a clinical and microbiologic response to chloramphenicol, respectively, but no corresponding decrease in deaths occurred (45). In our study, patients with urinary tract infections (UTI) (eight cases) were initially treated successfully with nitrofurantoin (100 mg/6 h). Ampicillin (12 g/day) was used in patients with infections other than UTI. In the latter group, however, the death rate was 33%, mostly because of severe sepsis. Chloramphenicol was not used in this group of patients. Although no controlled trials have demonstrated the effectiveness of chloramphenicol for the treatment of GRE, this antibiotic could be a therapeutic alternative in Colombia.

Linezolid, a new compound from the oxazolidinone group, has just been launched in Colombia; our findings indicate that it was active against all isolates tested. Linezolid has emerged as a therapeutic alternative for multiresistant GRE in Colombia, as in other parts of the world where it is currently available. However, linezolid-resistant *E. faecium* clinical isolates have already been reported in relation to long courses of therapy (21–40 days) (46). A linezolid-resistant *E. faecium* isolated from a patient without prior exposure to an oxazolidinone has also been described (47).

In this study, we report the first isolation and characterization of a multiresistant cluster of VanA-type *E. faecium* in a Colombian hospital. The emergence of this problem and the limitation of therapeutic options require the implementation of specific infection control measures and antibiotic policies to avoid further dissemination.
